# Single‐cell co‐expression analysis using computational machine learning reveals oxidative, immunopathologic, and myocardial responses for multi‐organ failure in COVID‐19

**DOI:** 10.1002/ctm2.1049

**Published:** 2022-10-06

**Authors:** Cheng Zhang, Ning Wang, Guoming Chen, Guoyi Tang, Chiwing Tam, Hor‐Yue Tan, Xiaoyu Xu, Yibin Feng

**Affiliations:** ^1^ School of Chinese Medicine Li Ka Shing Faculty of Medicine The University of Hong Kong Hong Kong China; ^2^ School of Chinese Medicine Hong Kong Baptist University Hong Kong China

Dear Editor,

Since more than half of the hospitalized coronavirus disease 2019 (COVID‐19) patients died of multi‐organ failure, it suggested severe challenges to COVID‐19 management in terms of currently limited knowledge.^1^ Herein, taking advantage of bulk RNA‐seq data (GSE162113 and GSE164805) and single‐cell RNA‐seq data (GSE165080), this study identified potential gene modules representing ‘Oxidative impairment’, ‘Immunopathological response’, and ‘Myocardial responses’ in COVID‐19 using R language programming. Also, drug candidates for multiorgan failure in COVID‐19 were indicated (Figure 1A).

1. Functional gene modules representing “oxidative impairment”, “immune‐pathological response”, and “myocardial dysfunction” in multi‐organ failure of COVID‐19 have been identified by single‐cell co‐expression analysis using machine learning
2. The pseudo time of FCGR3A^+^ monocytes to dendritic cells might be prior to FCGR3A^+^ monocytes‐derived pro‐inflammatory macrophage in COVID‐19.
3. FDA‐approved 20 medicines are potentially repurposed for COVID‐19 management

**FIGURE 1:**
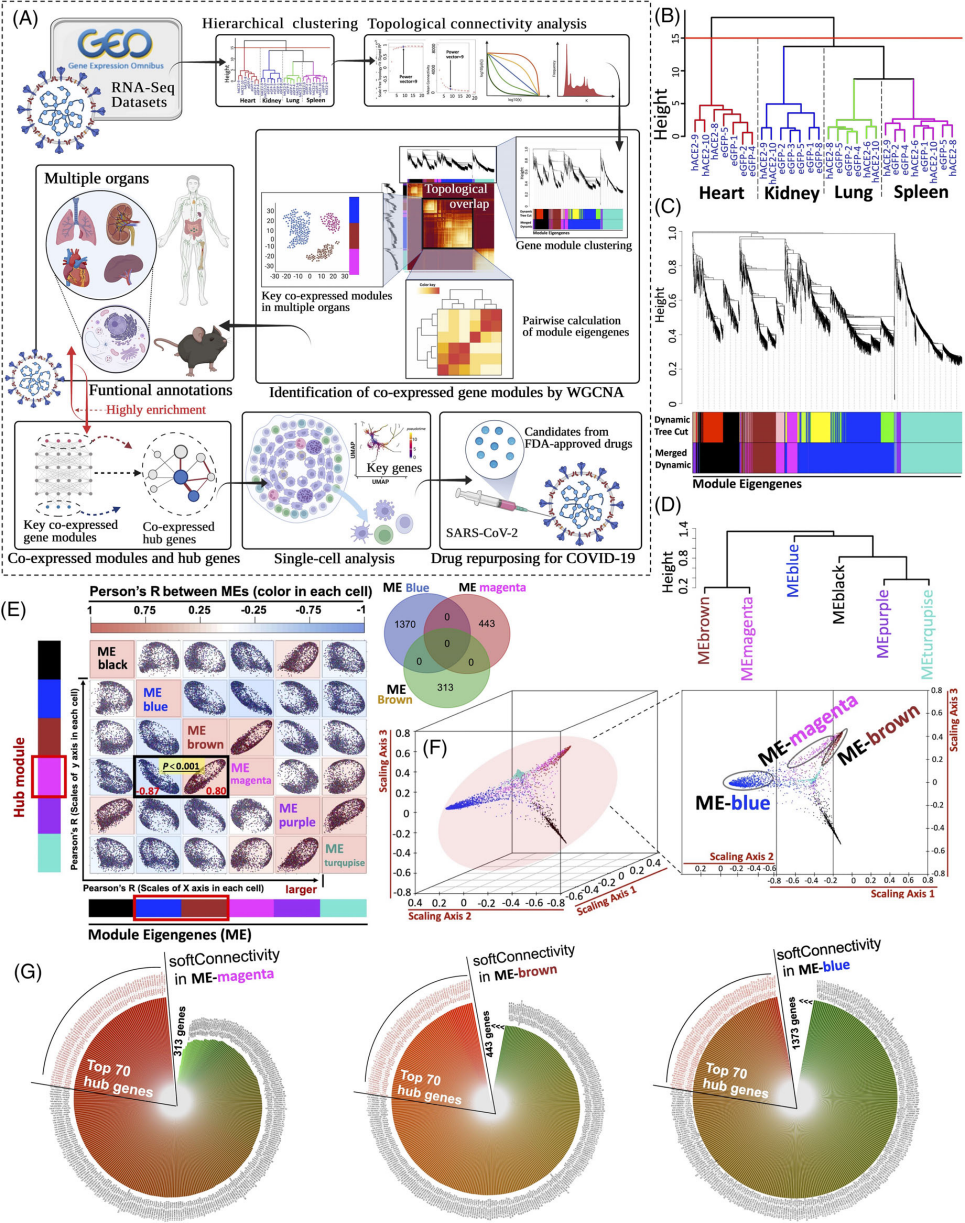
Co‐expression analysis for hub modules and genes for coronavirus disease 2019 (COVID‐19). (A) Schematic of single‐cell co‐expression analysis for gene modules and drug repurposing for COVID‐19. There are 41 bulk RNA‐seq samples (29 multi‐organs, Mus musculus; 12 pluripotent stem cells, Homo sapiens) from GSE162113; while 15 bulk RNA‐seq samples (peripheral blood mononuclear cell [PBMCs], Homo sapiens) were retrieved from GSE164805; Also, 55850 single‐cell RNA‐seq data were obtained from GSE165080 (B) Hierarchical clustering of genes in multiorgan samples (heart, kidney, lung, spleen). (C) Gene clustering dendrogram consists of hierarchical clustering and heatmap (adjacency‐based dissimilarity). The below various colours stand for the gene modules. (D) Highly‐correlated gene modules were merged in terms of the value of Module Eigengene. (E) A pairwise scatterplot of module eigengenes was in the left panel. The Venn diagram in the right panel represented the intermediate genes between modules. (F) Geometric interpretation of gene expression in 3D scatter plot. (G) Highly‐correlated hub genes (top 70) in modules ranked by Soft‐connectivity (ME‐magenta, ME‐brown, and ME‐blue)

For co‐expression analysis, using the R package WGCNA, those genes (3702 genes, Data S1) in GSE162113 with expression variances greater than the 90th percentile of the whole genome were involved in hierarchical clustering (Figure 1B).^2^ The scale‐free soft threshold was determined by the criteria of approximating scale‐free topology (Figure S1A). The co‐expressed genes among modules were shown in Figure 1C. Finally, 12 co‐expression modules were clustered into six modules (Figure S1B,D). The adjacency matrix‐based pairwise relationships among modules were shown in Figure S1C. In addition, we further quantified the correlation profiles between modules by calculating the Module Eigengene (ME)‐dependent Pearson's *r* and *p*‐value (Figure 1E), suggesting that ME‐magenta was strongly linked with ME‐blue and ME‐brown (*p* < .001 vs. ME‐blue or ME‐brown). Since intramodular genes cannot be the intermediate ones, the Venn (Figure 1E right panel) and 3D scatter plot (Figure 1F) indicated that genes in each module were independent of other modules. In Figure 1G, highly‐correlated key genes (Top 70) in these three modules were obtained in terms of intramodular soft‐connectivity analysed and visualised by “dplyr” with “ggplot2” (Data S4). Thus, ME‐magenta, ME‐blue, and ME‐brown may exert specific functions in COVID‐19 progression.

For functional analysis in gene modules, ME‐magenta were mainly enriched in the graphene oxide (GO)‐term 0055114: Oxidation‐reduction process “(*p* = 1.67 × 10^−32^)” and KEGG‐term “Hsa00190: Oxidative phosphorylation (*p* = 4.10 × 10^−56^)” (Figure 2A,D). The imbalance of oxidation‐reduction status may cause the disruption of the redox homeostasis and immune dysfunctions, which may result in higher susceptibility to severe acute respiratory syndrome coronavirus 2 (SARS‐CoV‐2) infection.^3^ Thus, ME‐magenta may classify as “Oxidative impairment in SARS‐CoV‐2”. As for the ME‐brown (Figure 2B,D), the enriched GO‐term are “0045214: Sarcomere organization (*p* = .0031)” and cardiac functions. While the KEGG‐term was “Hsa05412: Arrhythmogenic right ventricular cardiomyopathy (*p* = .0038)” and “Hsa05414: Dilated cardiomyopathy (*p* = .0083)”. These findings revealed the functional role of ME‐brown was “myocardial dysfunction”, a disease accounting for 60% of patients in hospitals with late‐stage COVID‐19.^4^ As for ME‐blue (Figure 2C,D), the enriched GO‐term was “0045944: Positive regulation of transcription from RNA polymerase II promoter (*p* = 2.8 × 10^−5^)”, while KEGG‐term was “Hsa04062: Chemokine signalling pathway (*p* = .0044)”. Herein, the functional profile of ME‐blue may be “immunopathological response in COVID‐19”. The most highly‐correlated genes (Top 10) in each module were shown in Figure 2E. Moreover, as shown in Figure S2A–C, the functional results (GO and KEGG terms) were almost identical to that of 70 genes in each module, showing the high representativeness of the Top 10 genes for each module (Supplementary Figure). Since the profile of peripheral blood mononuclear cell (PBMC) can reflect the host immune and oxidative responses in COVID‐19,^5^, ^6^ transcriptional profile in PBMCs with or without SARS‐CoV‐2 infection (GSE164805 Homo sapiens) was used for differential analysis in ME‐blue and ME‐magenta, showing that the damaged redox system is almost inversely proportional to autoimmune feedback (Figure 2F). On the other hand, in GSE162113, an RNA‐Seq dataset of cardiomyocytes originating from human pluripotent stem cells with or without COVID‐19 was used for differential analysis between ME‐magenta and ME‐brown, showing that the significant decrease of genes representing oxidative responses and cardiomyocytes in COVID‐19 patients (Figure 2G). Taken together, we identified key functional modules in COVID‐19 as follows: “Oxidative impairment (ME‐magenta)”, “Myocardial dysfunction (ME‐brown)”, and “Immunopathological response (ME‐blue)”, respectively.

**FIGURE 2:**
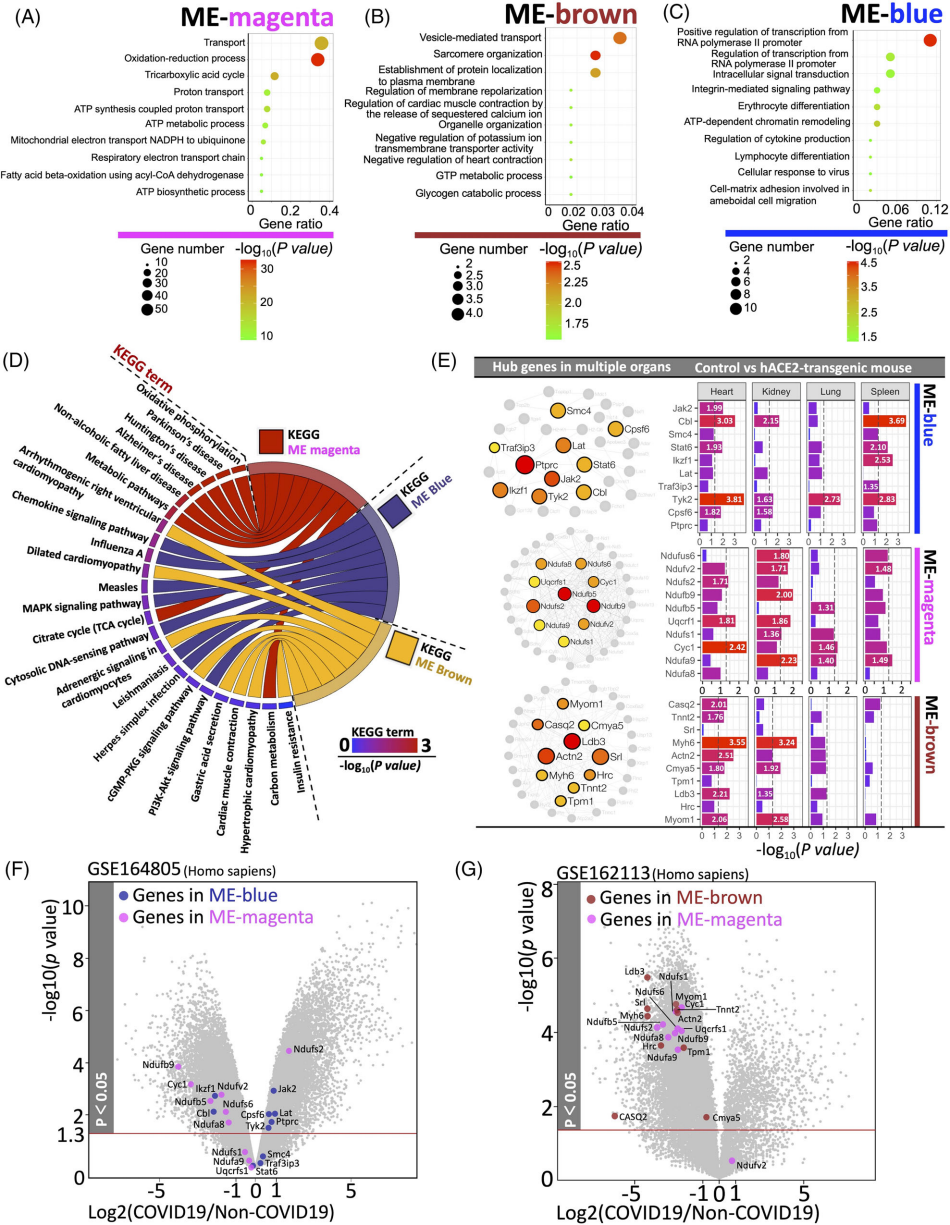
Functional and differential expression analysis of gene modules. (A–D) GO and KEGG functional enrichment analysis for genes (Top 70) in each module (ME‐magenta, ME‐brown, and ME‐blue). (E) Network and differential expressions (coronavirus disease 2019 [COVID‐19]/non‐COVID‐19) for the Top 10 co‐expressed hub genes in each module. (F, G) Volcano visualization represents the differential expressions (COVID‐19/non‐COVID‐19) and correlation analysis of hub genes in Homo sapiens datasets (GSE164805 and GSE162113)

Compared with bulk RNA‐seq, single‐cell RNA‐Seq analysis using Seurat could point out rare cell classifications and clarify transitions of cell states at different developmental stages. Therefore, we performed a single‐cell analysis from PBMCs in COVID‐19 patients (GSE165080). In a Uniform Manifold Approximation and Projection (UMAP) plot, 12 cell types were identified (Figure 3A). In addition, protein tyrosine phosphatase receptor type C (PTPRC) is an essential regulator of antigen receptors of T cells, B cells, and immunological synapses.^7^ In the right panel of Figure 3A, PTPRC was comprehensively and intensively expressed in distinct immune cells, suggesting a key target candidate for COVID‐19 (Figure 3B–D). For cell chat analysis using the R package CellChat, both “macrophage migration inhibitory factor (MIF) pathway” and “type II interferon (IFN‐II) signalling pathway” are the main regulatory pathways in COVID‐19, in which CD8+ T cell is the most high‐influence cell signal sender (Figure 3E). For cellular developmental trajectories among immunocytes, Monecle3 was used to conduct psedotemporal ordering of PBMCs. It is reported that severe COVID‐19 can result in monocyte dysfunction followed by increasing monocyte‐derived inflammatory macrophages and decreasing monocyte‐derived dendritic cells.^8^ However, there is no report on the evolutional time and relative order of this process. Hereby, we explore the dynamics of pseudotime trajectory in cell types shown in the UMAPs (Figure 3F), indicating that the pseudotime of FCGR3A+ monocytes to dendritic cells (Number 14) may be prior to FCGR3A+ monocytes‐derived pro‐inflammatory macrophage with high expression of IL1β (Number 18) (Data S2). In addition, the activation of B and T cells (CD4+ T, CD8+ T, and Memory T cells) may involve in the immune response of PTPRC in COVID‐19 (Figure 3G). Thus, the single‐cell analysis may reflect the regulatory immune landscape of ME‐blue in COVID‐19. The proposed pathological mechanism of the top 10 genes in gene modules (ME‐magenta, ME‐brown, and ME‐blue) were shown in Figure 4A. Furthermore, based on identified genes and DGldb,^9^ 20 Food and Drug Administration (FDA)‐approved drugs were identified for potential COVID‐19 management (Data S5 and Figure 4B). For instance, Baricitinib (JAK inhibitor) with Remdesivir can accelerate the recovery of hospitalised patients with COVID‐19.^10^ Since the pharmacological targets and mechanism of FDA‐approved medicines are clear, it is attractive to further validate these candidates for COVID‐19 therapy. More detailed descriptions regarding the methods and understanding of identified targets were shown in Data S3.

**FIGURE 3:**
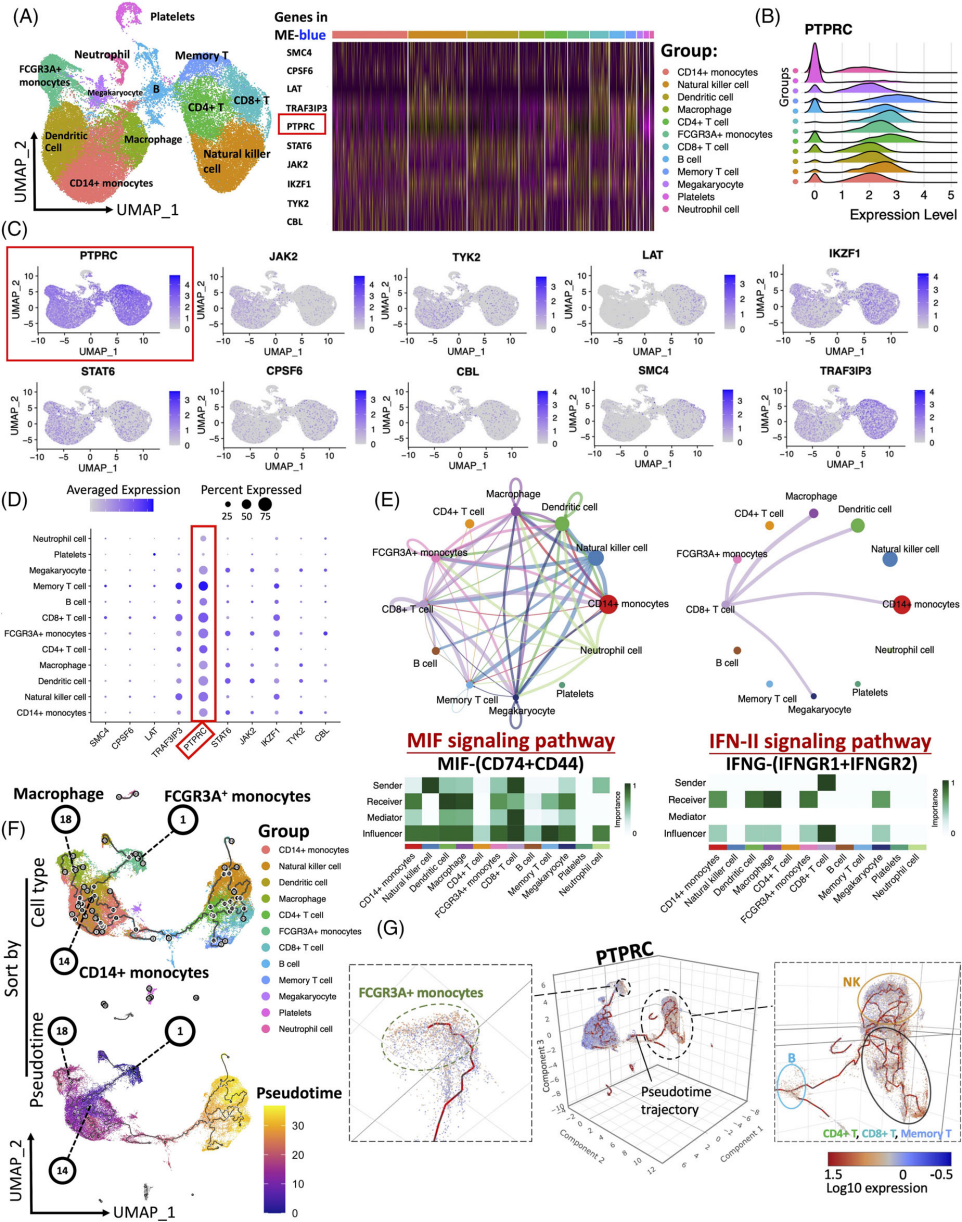
Single‐cell analysis for genes from peripheral blood mononuclear cells (PBMCs) of coronavirus disease 2019 (COVID‐19) patients. (A) A UMAP analysis of 55850 cells from 42 COVID‐19 patients in various cell types (left panel), including CD14+ monocytes (VCAN, CD14, LYZ), Natural killer cell (GNLY, NKG7), Dendritic cell (CD83, TYMP), Macrophage (CD68, CD163, IL1B), CD4+ T cell (CD4, CD3D, CD3E), FCGR3A+ monocytes (FCGR3A, CD68, MS4A7), CD8+ T cell (CD8A, CD3D, CD3E), B cell (MS4A1, CD19, CD79A, Memory T cell (IL7R, LTB, CD3D, CD3E), Megakaryocyte (PPBP, NRGN), Platelets (PPBP, GP9, ITGA2B), Neutrophil cell (CD177, LYZ). A heatmap of key gene expression in ME‐blue on a principal component (right panel). (B) The ridge plot of protein tyrosine phosphatase receptor type C (PTPRC) expression across cell types. (C) A UMAP‐related feature plot for genes in ME‐blue. (D) A dotplot showing the feature expression of genes in ME‐blue across cell types. (E) Cell‐cell communication atlas in MIF and type II interferon (IFN‐II) pathways. (F) Constructing trajectories in cell populations sorted by cell type and pseudotime. (G) Pseudotime trajectory in cell populations expressing PTPRC

**FIGURE 4:**
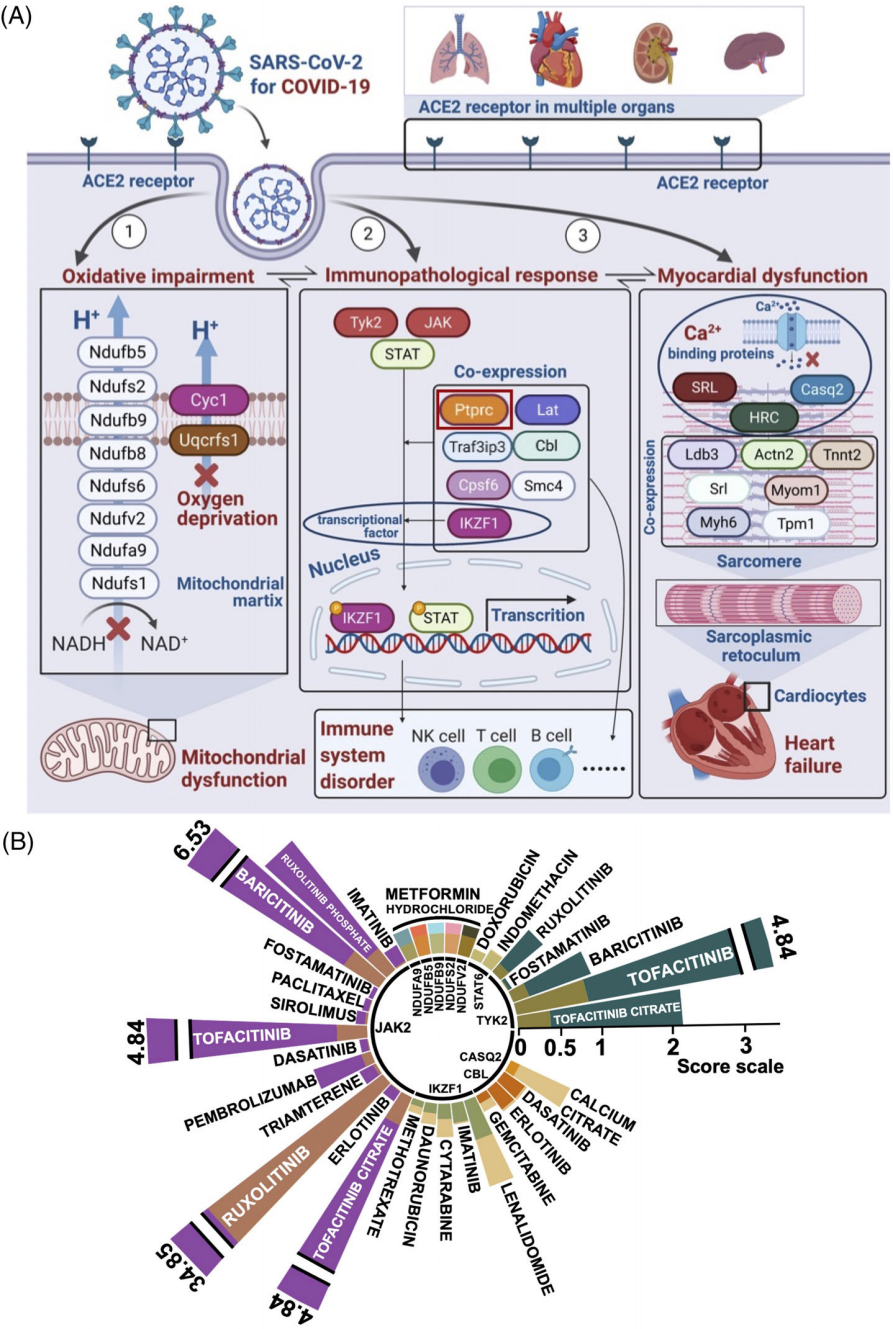
The proposed gene module‐related pathological mechanism and drug repurposing for coronavirus disease 2019 (COVID‐19). (A) The estimated pathological mechanisms of the top 10 genes in gene modules (ME‐magenta, ME‐brown, and ME‐blue) in COVID‐19. (B) Food and Drug Administration (FDA)‐approved medicines as candidates for drug repositioning against COVID‐19. The value of the Interaction Score mainly depends on the evidence from publications (brown columns), while Query Score (various colours except brown in columns) represents the specific relationship between the given drugs with genes. The larger value of both Interaction Score and Query Score points to more possibility of the FDA‐approved drug for the corresponding targets

In conclusion, the main merits of this study are as follows: 1) Using single‐cell co‐expression analysis, we identified functional gene modules representing “oxidative impairment”, “immunopathological response”, and “myocardial dysfunction” in multi‐organ failure of COVID‐19, which may promote COVID‐19 management. 2) Based on computational machine learning analysis, the pseudo time of FCGR3A^+^ monocytes to dendritic cells might be prior to FCGR3A^+^ monocytes‐derived pro‐inflammatory macrophage in COVID‐19, showing the therapeutic strategy of COVID‐19. 3) FDA‐approved 20 medicines are potentially repurposed for COVID‐19 management.

## CONFLICT OF INTEREST

The authors declare that they have no conflict of interest.

## Supporting information



Supporting Information

Supporting Information

Supporting Information

Supporting Information

Supporting Information

Supporting Information
