# Supplementary material for: Single‐cell co‐expression analysis using computational machine learning reveals oxidative, immunopathologic, and myocardial responses for multi‐organ failure in COVID‐19
Source: Clin Transl Med. 2022 Oct 6;12(10):e1049. doi: 10.1002/ctm2.1049 (PMC9538710; doi:10.1002/ctm2.1049)
Supplement: Supplementary file 1 — Supporting Information [file CTM2-12-e1049-s006.docx]

**Supplementary Figures:**


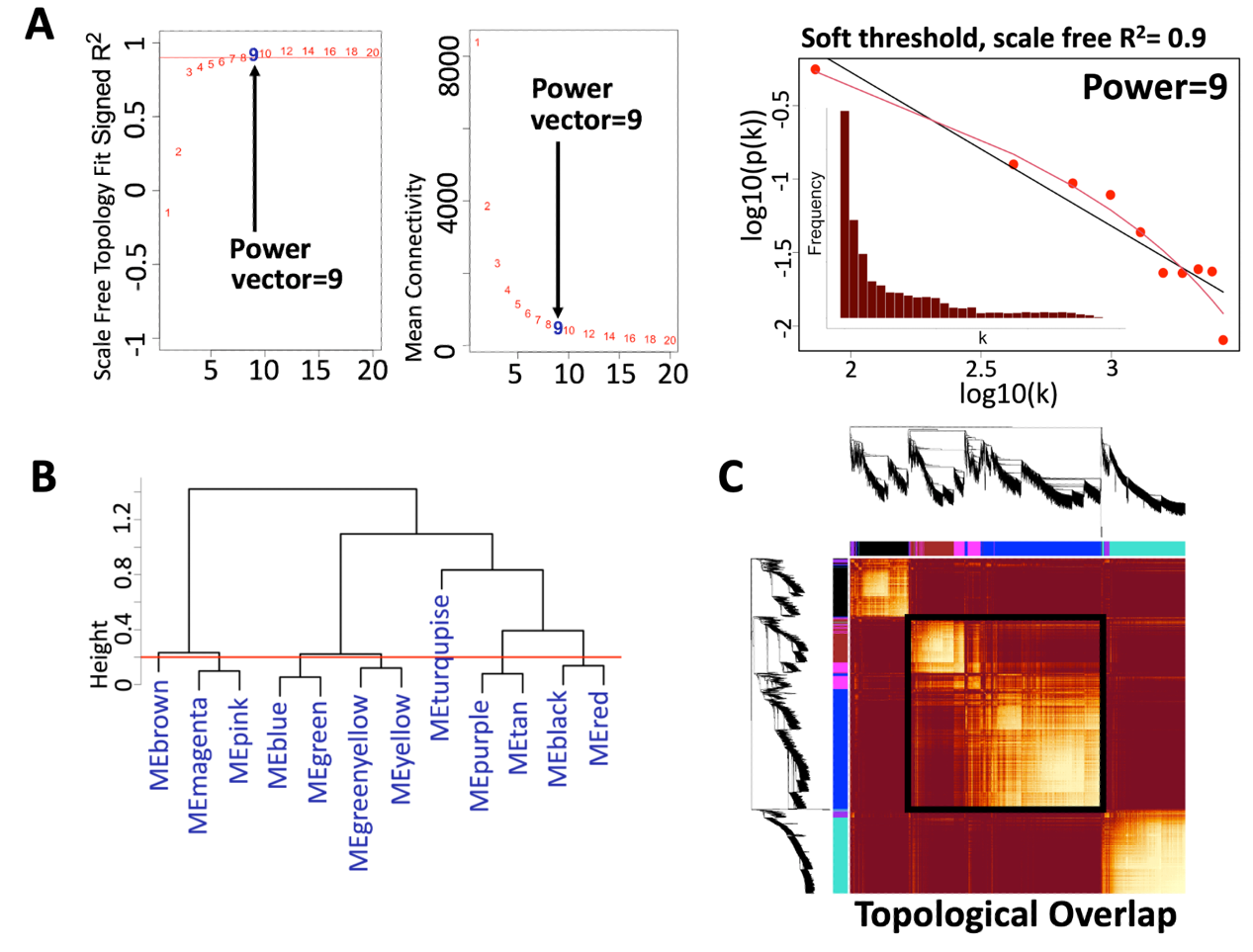


**Figure S1: Hierarchical clustering and topological connectivity analysis.**

(A) Analysis of network topology for powers of soft threshold. The left panel indicates the index of scale-free topology fit (y-axis) as a function of the soft-thresholding power (x-axis). The middle panel shows value of mean connectivity (y-axis) as a function of soft-thresholding power (x-axis). The right panel indicates a log-log plot of connectivity of signed adjacency matrices. (B) Highly-correlated gene modules in terms of the value of Module Eigengene (cur-off height=0.2). (C) Topological overlap plot of filtered genes (variances larger than 90th percentile of the whole genome). Progressively darker color represents higher overlap

**
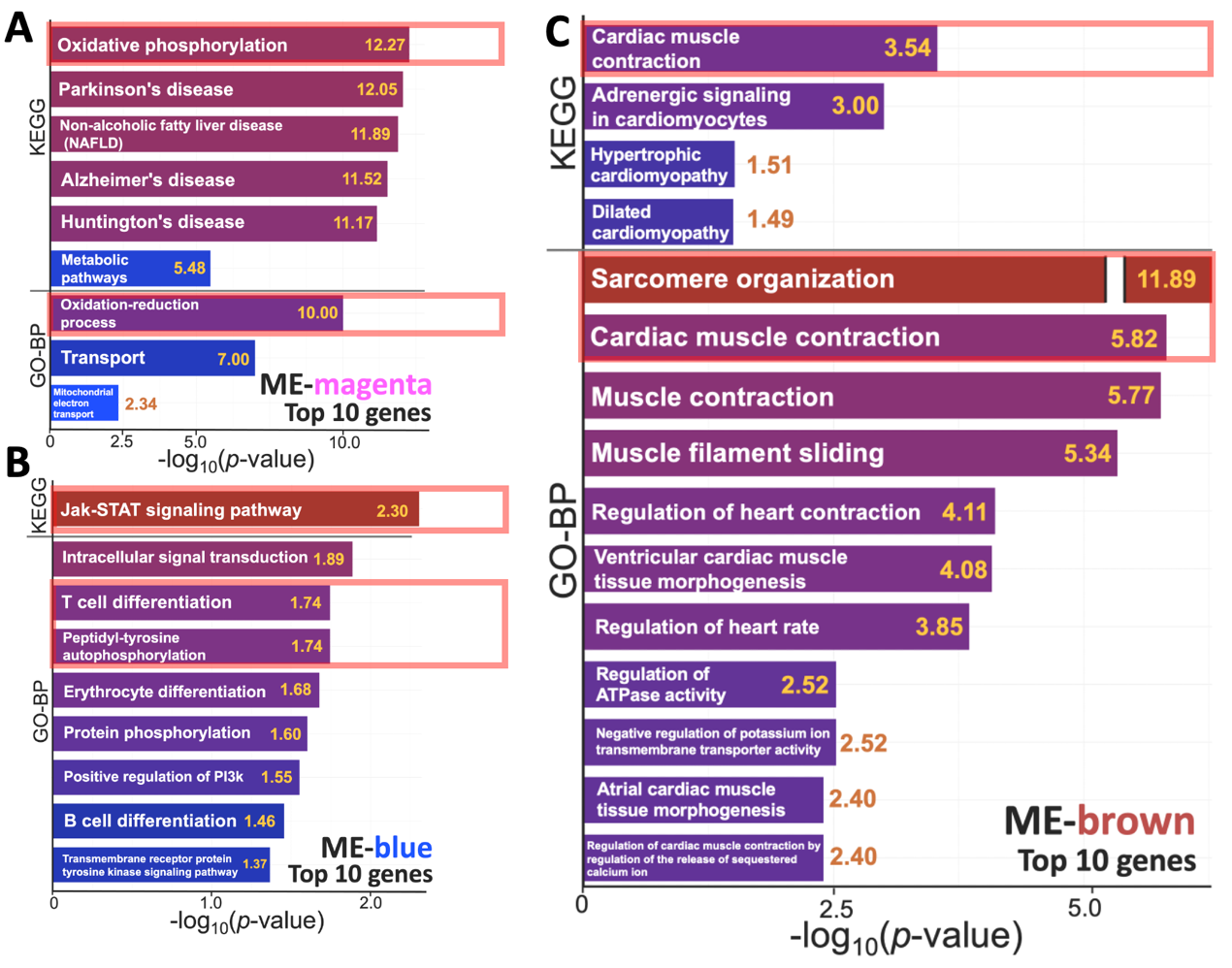
**

Figure S2: Highly representative functional analysis (GO-term, KEGG-term) of Top 10 intramodular genes in each module (ME-magenta, ME-brown, ME-blue).
