# Supplementary material for: Single‐cell co‐expression analysis using computational machine learning reveals oxidative, immunopathologic, and myocardial responses for multi‐organ failure in COVID‐19
Source: Clin Transl Med. 2022 Oct 6;12(10):e1049. doi: 10.1002/ctm2.1049 (PMC9538710; doi:10.1002/ctm2.1049)
Supplement: Supplementary file 2 — Supporting Information [file CTM2-12-e1049-s002.docx]

**Supplementary Data 1**

**1. Methods**

1.1. Data acquisition for WGCNA-related co-expression analysis

Both GSE162113 and GSE164805 were downloaded from NCBI- GEO database for co-expression analysis ^1,2^. As for GSE162113 (Mus musculus part), those hACE2 transgenic mice undergoing 7-day SARS-CoV-2 infection were included in our study. Ultimately, 37017 genes in 29 samples were acquired from heterogeneous organs for further analysis, including heart, kidney, lung, and spleen. Additionally, both GSE162113 (Homo sapiens part) and GSE164805 were used to validate the correlations between gene modules in human datasets. In detail, GSE162113 (Homo sapiens part) consists 12 human pluripotent stem cell (hpsc)-derived cardiomyocytes with or without 24- or 48-hour SARS-CoV-2 treatment were selected. Notably, since circulating PBMC cells are responsible for building blood cells in the immune system, the alternations in PBMC may demonstrate the fluctuations of host immune responses of multiple organs during SARS-CoV-2 infection. Thus, 15 human peripheral blood mononuclear cells (PBMCs) samples from severe COVID19 patients and healthy controls in GSE164805 were included.

1.2. Data filtering for WGCNA

For data processing, the function “apply ()" in R was used for ranking the variance of gene expressions in hACE2 transgenic mice with or without SARS-CoV-2 infection. Since non-varying genes are commonly noisy data with disproportionately low expression in COVID-19 or non-COVID-19 groups, those expression variances greater than 90^th^ percentile of the whole genome was exported for further study. The missing or zero-variance gene expression with correlation strengths below a threshold (verbose=3, minRelativeWeight=0.1 in R) was excluded. This process was circularly repeated until the enrolled genes are stable. Afterwards, 3702 of 37017 genes were left as the most informative ones for WGCNA analysis. Of note, the method of filtering genes by significantly differential expressions are not applied in this study. Because differential gene-based analysis usually fails to establish gene modules and invalidate the scale-free topology assumption.

1.3 Sample clustering and WGCNA-related gene module construction

For sample clustering, hierarchical cluster analysis of samples from various organs was processed by the function “hclust ()” in R package STATS. Since cluster structure is vulnerable to outliers, sample data profile irrelevant to any clusters was removed according to the Euclidean distance in the clustering dendrogram. Based on the intra-cluster similarity of transcriptional expressions, homogeneous sample microarray from multiorgan will be grouped in a consensus branch site.

For the construction of gene modules, firstly, the similarity of the consensus matrix was identified by calculating the coefficients of Pearson’s correlation sij= cor(i, j) of the whole gene set. It was further converted into a weighted adjacency matrix for enhancing the co-expression similarity by the following equation: a_ij_ = | (1+cor(i, j))/2 |^β^, where β represents the soft thresholding power and a_ij_ points to the resulting adjacency accessing the strength of weighted connectivity. Notably, the power β (threshold parameter for weighted analysis) is chosen by the criteria of approximating scale-free topology by the R function “pickSoftThreshold ()” in WGCNA package. Thus, β=9 meets the criteria for establishing scale-free network, which flattens out upon reaching both the value of scale-free topology fit signed R^2^ (R^2^=0.9 in this study) and the minimum value of mean connectivity. Afterwards, a topological overlap matrix (TOM) was carried out from the adjacency matrix. TOM was in-turn transformed to a dissimilarity TOM. Then, hierarchical clustering was used to produce a dendrogram of classifying the consensus profile of gene expression in a module. Using the R function “plotDendroAndColors()” in WGCNA package, all the modules are established by a dynamic tree-cutting algorithm followed by merging the close-distance modules upon the calculation and clustering of Module Eigengene values (cut-off height=0.2). For the value of Module Eigengene (ME), it is based on the value of the first principal component of gene expression matrix of the corresponding gene modules analyzed by principal component analysis (PCA), which is calculated by R function “signedKME()” in WGCNA package. The higher the absolute value of MM, the greater the correlation degree between genes and modules. In particular, the ranging value of MM is from -1 to 1, suggesting a positive or negative correlation, respectively. Specifically, MM=0 indicates that genes are not associated with a specific module.

1.4. Identification of consensus gene module and hub genes

For the identification of gene modules with co-expressions, to begin with, TOM-related weighted heatmap was plotted according to the dissimilarity of gene expressions. It was visualized by the R function “TOMplot()” in WGCNA package. In a TOM heatmap of our study, each row or column was pointed to a single gene. Modules corresponding to branches of gene dendrograms were illustrated at the top or left side of the heatmap. Blocks of gene expression with progressively darker or lighter colors indicated the low or high adjacency, respectively. To further analyze the relationships among various modules, the value of Module Eigengene were adopted as representative profiles for quantifying module similarity. Both intermodular Pearson’s r and p-value provided by Kruskal-Wallis test and one-way ANOVA with Tukey’s multiple comparison test were calculated for measuring eigengene correlation. The smaller p-value represented the more significant intermodular eigengenes connectivity. Highly relevant modules were defined when a p-value for the eigengene correlation was less than 0.05. These processes relied on the basic function in R. Alternatively, the relationships among modules can be illustrated by a multi-dimensional view, such as a 3D scatter plot by R. For hub genes identification, it can be performed by the determination of soft-thresholding power (gene connectivity in a weighted adjacency matrix) by WGCNA. The sum of its adjacency can calculate soft connectivity. Thus, highly-connected genes of co-expression modules can be considered as regulator modules for COVID-19. The visualization of key genes is shown in the Nightingale’s rose map by R package “dplyr” with “ggplot2”.

To further screen the most predominant genes for a module, Cytoscape, an approach for network visualization and analysis, was used to interpret the gene-gene interaction by plug-in functions, including StringApp and cytoHubba. In brief, the add-on stringApp imports functional protein-protein association networks from the database STRING. Selected proteins were encoded by the identified hub genes. STRING is equipped with a series of functional classifications, such as GO and KEGG. After the construction of network, cytoHubba in Cytoscape further prioritized genes with high soft connectivity in terms of a topological algorithm (gene-gene connectivity). Therefore, the most central and contributive genes within each module were selected for the anti-SARS-CoV-2 drug repositioning and gene validation

1.5 Differential gene analysis for co-expression gene modules

Differential gene analysis was applied to measure the differential expression and correlation between gene modules in groups with or without SARS-CoV-2 infection in GSE162113 and GSE 164805. Pearson’s product-moment correlation test was used to evaluate the module relationship upon gene differential expressions.

1.6 Single-cell clustering signature and cell-type annotation

For an in-depth understanding of the genetic profile of ME-blue responsible for “immunopathological response in COVID-19”, we reanalyzed a COVID19-related single-cell dataset GSE165080 from NCBI GEO^3^. The information of 42 COVID-19 patients with 181,562 cells was used from this dataset, which is previously established using 10x Genomics. For the quality control, 55,850 cells were finally collected in terms of the threshold as the following: gene number in each cell ranging from 200-2500; percentage of mitochondrial DNA-derived gene (< 20%), haemoglobin-related gene (< 5%), and ribosome-associated gene (< 20%). Afterwards, the Seurat function (version 4.1.0) was adopted for normalization (DESeq2 function), dimensional reduction and, cell population clustering (PCA, UMAP, and weighted shared nearest neighbor (SNN) function). PanglaoDB-related gene annotation was used for labelling the specific cell population, including CD14+ monocytes (CD14, LYZ), Natural killer cell (GNLY, NKG7), Dendritic cell (CD83, TYMP), Macrophage (CD68, CD163, IL1B), CD4+ T cell (CD4, CD3D, CD3E), FCGR3A+ monocytes (FCGR3A, CD68), CD8+ T cell (CD8A, CD3D, CD3E), B cell (MS4A1, CD19, CD79A, Memory T cell (CCR7, CD3D, CD3E), Megakaryocyte (PPBP, NRGN), Platelets (PPBP, GP9, ITGA2B), Neutrophil cell (CD177, LYZ). The result visualization was performed by UMAP, heatmap, ridge, and dot plots. The cross-talks among immunocytes are determined by the “CellChat” package in R.

1.7 Trajectory inference in single-cell population with Monocle 3

The Monocle3 was used to clarify the role of key genes from the immune response module (ME-blue) in developmental trajectories of cell types (e.g. from monocytes to dendritic cells) in COVID-19. The analytic process of Monocle 3 is mainly composed of 4 steps for organizing cells into potentially discontinuous trajectories ^4^. It includes 1) Dimensionality reduction with UMAP. 2) Partitioning cells into discontinuous trajectories; 3) Studying cellular developmental progressions using SimplePPT. 4) Identifying genes with trajectory-dependent expression using Moran’s I test.

1.8 Predicting drug repositioning against SARS-CoV-2

The Drug-Gene Interaction Database (DGldb, <http://dgidb.org/>) was adopted to provide information on potentially druggable genes according to publications and databases ^5^. FDA-approved medicines for specific hub genes were screened out in the DGIdb database, which aimed to predict repositioned drugs for COVID-19 therapy.

**2. Discussion:**

In this study, we identified key co-expression gene modules classified as “oxidative impairment (ME-magenta)”, “immunopathological response (ME-blue)”, and “myocardial dysfunction (ME-brown)” respectively, which may involve in multi-organ failure of COVID-19. As for ME-magenta (oxidative impairment), 8 of the 10 genes were members of NADH dehydrogenase^6^. Surprisingly, representative genes for oxidative impairment in multi-organs and cross-platform species were downregulated after SARS-CoV-2 infection, indicating oxygen deprivation in COVID-19 progression. Moreover, deficiency of NADH dehydrogenases is widespread in populations with hypertension, diabetes and obesity, of which groups are highly susceptible to SARS-CoV-2 exposure^7^. Hereby, the reduction of identified 8 co-expression NADH dehydrogenases may alleviate systematic complications and sensitize host cells to mitochondrial respiration dysfunction in COVID-19. For Cyc1 and Uqcrfs1 in ME-magenta, both are subunits of cytochrome bc1 complex and engaged in the biochemical generation of ATP^8^. Repression of CYC1 and UQCRFS1 can impair ATP synthesis^9^. ATP-related energy deficiency is an aggressive pathological response in severe COVID-19 due to unmanageable energy budget^10^. Thereby, our study raises the possibility that mediating 10 hub genes in ME-magenta may specifically alleviate oxidative impairment in COVID-19 patients.

In COVID-19, ACE2 can be regulated by immunocytes, including monocytes, macrophages, lymphocytes, NK cells, B cells, and T cells. Additionally, JAK-STAT pathway is the downstream targets of ACE2^11^. Consistent with our findings, “JAK-STAT signaling pathway" combined with “T cell or B cell differentiation” was the most enriched functional annotations in ME-blue, suggesting the potential immunomodulatory role of JAK-STAT in COVID-19. It is noteworthy that interferons (IFNs) are broad-spectrum antiviral proteins. IFNs can activate interferon-stimulated genes (ISGs) by modulating JAK-STAT pathway^12^. Notably, it is reported that severe SARS-CoV-2 infection was associated with Type I IFN deficiency^13^. Given a wide distribution of Type I IFN receptors in organs, the increase of JAK-STAT pathway may reverse multiorgan failure in COVID-19. For ME-blue, PTPRC is one of the top 10 co-expressed genes in ME-blue. Protein tyrosine phosphatase is an essential regulator of antigen receptor of T cells, B cells, and immunological synapses ^14^. As a result of single-cell analysis in PBMCs of COVID-19 patients, PTPRC was widely expressed in multiple immunocytes (Figure 6C), especially in B and T cells (CD4+ T, CD8+ T, Memory T), suggesting the activation of lymphocyte responses in COVID-19 progression. As the substrates of PTPRC, JAK kinases can be regulated by PTPRC, indicating the potential anti-COVID-19 action of PTPRC ^15^. Ikzf1 is pivotal for lymphopoiesis and known as a transcription factor for hematopoietic and immunocytes, such as B cell, CD4^+^ T cells^16^. Dysfunction of Ikzf1 is linked with B cell apoptosis, lymphoproliferative disorder, and failure in the differentiation of B cells or T helper cells^17,18^. Mutation of Cbl, an E3 ubiquitin-protein ligase, is implicated in myeloid malignancies, which may impair T, B, NK cell immunity^19^. TRAF3IP3, a Golgi-related target, is a vital mediator of thymocyte growth. Insufficient TRAF3IP3 could attenuate the formation of mature thymocytes ^20^. Lat, as known as the linker of activated T cells, is essential for T cell anergy^21^. CPSF6 is a subunit of the cleavage factor I complex for RNA cleavage and polyadenylation processing. It is reported that cytoplasmic CPSF6 can inhibit innate immunity for HIV-1 nucleic acids in monocyte-derived macrophages^22^. Whether CPSF6 helps SARS-CoV-2 escaping from the host immune responses remains to disclose. Smc4, a core subunit of condensin, can enhance the production of Type I IFN-β in innate response, suggesting that Smc4-induced IFN-β overexpression may aggravate SARS-CoV-2 infection^23^. Moreover, as results of single-cell analysis in ME-blue, both MIF and IFN-II pathways were identified as key cell communication signalling in COVID-19. It was reported that activation of either IFN-II (IFN-gamma) and MIF factor was the hallmark of severe COVID-19 ^24,25^. Our CellChat analysis also validated these results. Furthermore, targeting MIF-CD74/CD44 in MIF pathway and IFNG-IFNGR1/IFNGR2 in IFN-II pathway may be the novel therapeutic targets of severe COVID-19. Based on developmental trajectories analysis, it suggested that FCGR3A+ monocytes may prefer to become dendritic cells rather than pro-inflammatory macrophages. Taken together, the measures of abolishing immune system dysfunction in COVID-19 may be the followings: 1) Targeting PTPRC-related co-expression genes (especially in ME-blue) for COVID-19; 2) Regulating MIF (MIF-CD74/CD44), IFNI (JAK-STAT), IFN-II (IFNG-IFNGR1/IFGNR2) pathway may improve immune microenvironment in COVID-19; 3) Reverse of potential FCGR3A+ monocytes-derived pro-inflammatory macrophages may inhibit COVID-19.

ACE2 receptors are broadly distributed in cardiopulmonary tissues, in which SARS-CoV-2-induced cardiovascular comorbidities are high mortality with poor prognosis^26^. Moreover, cardiac sarcomeres are not only responsible for blood ejection but also engaged in signal transduction^27^. Therefore, it is urgent to disclose the pathogenesis of myocardial disorder in COVID-19. In our study, ME-brown points to “Cardiac muscle contraction” and “Sarcomere organization”, suggesting the role of “Myocardial dysfunction” in COVID-19. SARS-CoV-2 can provoke destabilizing of the cardiac contractile system in the advanced stage of SARS-CoV-2 infection. Consistent with our findings, the 9 hub genes (Ldb3, Actn2, Tnnt2, Srl, Casq2, Myom1, Myh6, Hrc, and Tpm1) may play a role in abnormal myocardial contraction^28^. More specifically, aberrant expressions of Actn2, Tnnt2, Myh6, Myom1 are potentially correlated with hypertrophic and dilated cardiomyopathy, in which Myh6 is abundant in late-onset of hypertrophic/dilated cardiomyopathy^29,30^. In addition, Ca^2+^ binding proteins, including SRL, Casq2, and HRC, are mainly expressed in the sarcoplasmic reticulum^31^. Among these, SRL overexpression can lead to cardiac muscle fatigue by impairing store-operated calcium entry^32^. Mutation of cardiac CASQ2 is linked with cardiac arrhythmia, catecholaminergic polymorphic ventricular tachycardia, and even sudden death^33^. HRC dysfunction is associated with abnormal releasing Ca^2+^ into sarcoplasmic reticulum, which may disturb cardiac contractile function^34^. These data provide the potential rationale for SARS-CoV-2-induced impairment of Ca^2+^ homeostasis in myocardial cells. Previous reports indicated that severe COVID-19 is accompanied by arrhythmia^35^. Both overexpression of Actn2 and Casq2 can result in cardiac arrhythmia, suggesting the potential involvement of SARS-CoV-2 infection^36^. Additionally, as an anchoring protein in suppressing calcineurin-mediated transcriptional activity, Cmya5 mutation comprises cardiac muscular dystrophy, suggesting the potential weakness of cardiac skeletal muscles induced by SARS-CoV-2 infection^37^. Therefore, based on the findings, these 10 hub genes related to pathogenic cardiac phenotype may serve as novel therapeutic targets in combating COVID-19. Of note, gene module “oxidative impairment (ME-magenta)” is strongly correlated with “immunopathological response (ME-blue)” or “myocardial dysfunction” (Magenta *v.s.* Blue p=0.0012; Magenta *v.s.* Brown p=2.49*10^-6^), suggesting that simultaneous regulation of specific intermodular biomolecules may be effective for COVID-19 management.

To provide therapeutic strategies to COVID-19, we performed drug repurposing according to drug-hub gene interaction from U.S. FDA database. Among these candidates, Baricitinib (JAK inhibitor), in combination with Remdesivir (SARS-CoV-2 RNA-dependent RNA polymerase inhibitor) can shorten the recovery period of hospitalized COVID-19 patients. Meanwhile, Tofacitinib and Fostamatinib are undergoing clinical trials for testing the alleviation of COVID-19-relevant pulmonary complications in ospitalized patients (Tofacitinib-NCT04469114; Fostamatinib-NCT04579393). In addition, Metformin was reported to suppress SARS-CoV-2-induced mortality in Type II diabetic patients by cytokine-reduction and immunomodulatory action^38^. Therefore, further investigations of these 20 candidates may be cost-effective and promising for timely controlling SARS-CoV-2 infection. Additionally, for our actions against COVID-19 in Hong Kong, we aim to explore Chinese medicine to reduce COVID-19 susceptible populations in a randomized, double-blinded clinical trial (NCT04668222). In terms of Chinese medicine theory, we selected herbal formula includes “Yu-Ping-Feng (YPF) formula”, “Xiang-Sha-Liu-Jun (XSLJ) formula”, “Liu-Wei-Di-Huang (LWDH) formula”. More specifically, YPF is documented for recovering redox homeostasis (primarily by repairing oxidized base)^39^, while LWDH is involved in immunomodulation (mainly by increasing IFN-γ) ^40^, and XSLJ is related with reducing inflammatory reaction^41^. Further experimental validations are indispensable to provide more scientific evidence for herbal medicine-related COVID-19 management.

Reference

1. Li S, Ma F, Yokota T, et al. Metabolic reprogramming and epigenetic changes of vital organs in SARS-CoV-2-induced systemic toxicity. *JCI Insight*. Jan 25 2021;6(2)doi:10.1172/jci.insight.145027

2. Zhang Q, Meng Y, Wang K, et al. Inflammation and Antiviral Immune Response Associated With Severe Progression of COVID-19. Original Research. *Frontiers in Immunology*. 2021-February-18 2021;12(135)doi:10.3389/fimmu.2021.631226

3. Wang X, Bai H, Ma J, et al. Identification of Distinct Immune Cell Subsets Associated With Asymptomatic Infection, Disease Severity, and Viral Persistence in COVID-19 Patients. *Front Immunol*. 2022;13:812514. doi:10.3389/fimmu.2022.812514

4. Cao J, Spielmann M, Qiu X, et al. The single-cell transcriptional landscape of mammalian organogenesis. *Nature*. Feb 2019;566(7745):496-502. doi:10.1038/s41586-019-0969-x

5. Cotto KC, Wagner AH, Feng YY, et al. DGIdb 3.0: a redesign and expansion of the drug-gene interaction database. *Nucleic Acids Res*. Jan 4 2018;46(D1):D1068-D1073. doi:10.1093/nar/gkx1143

6. Nassar OM, Wong KY, Lynch GC, Smith TJ, Pettitt BM. Allosteric discrimination at the NADH/ADP regulatory site of glutamate dehydrogenase. *Protein Sci*. Dec 2019;28(12):2080-2088. doi:10.1002/pro.3748

7. Miller R, Wentzel AR, Richards GA. COVID-19: NAD(+) deficiency may predispose the aged, obese and type2 diabetics to mortality through its effect on SIRT1 activity. *Med Hypotheses*. Nov 2020;144:110044. doi:10.1016/j.mehy.2020.110044

8. Smith PM, Fox JL, Winge DR. Biogenesis of the cytochrome bc(1) complex and role of assembly factors. *Biochim Biophys Acta*. Feb 2012;1817(2):276-86. doi:10.1016/j.bbabio.2011.11.009

9. Rocha S, Freitas A, Guimaraes SC, Vitorino R, Aroso M, Gomez-Lazaro M. Biological Implications of Differential Expression of Mitochondrial-Shaping Proteins in Parkinson's Disease. *Antioxidants (Basel)*. Dec 21 2017;7(1)doi:10.3390/antiox7010001

10. Ozilgen M, Yilmaz B. COVID-19 disease causes an energy supply deficit in a patient. *Int J Energy Res*. Sep 29 2020;doi:10.1002/er.5883

11. Luo J, Lu S, Yu M, et al. The potential involvement of JAK-STAT signaling pathway in the COVID-19 infection assisted by ACE2. *Gene*. Feb 5 2021;768:145325. doi:10.1016/j.gene.2020.145325

12. Majoros A, Platanitis E, Kernbauer-Holzl E, Rosebrock F, Muller M, Decker T. Canonical and Non-Canonical Aspects of JAK-STAT Signaling: Lessons from Interferons for Cytokine Responses. *Front Immunol*. 2017;8:29. doi:10.3389/fimmu.2017.00029

13. Schreiber G. The Role of Type I Interferons in the Pathogenesis and Treatment of COVID-19. *Front Immunol*. 2020;11:595739. doi:10.3389/fimmu.2020.595739

14. Castro-Sanchez P, Aguilar-Sopena O. Regulation of CD4(+) T Cell Signaling and Immunological Synapse by Protein Tyrosine Phosphatases: Molecular Mechanisms in Autoimmunity. *Front Immunol*. 2019;10:1447. doi:10.3389/fimmu.2019.01447

15. Porcu M, Kleppe M, Gianfelici V, et al. Mutation of the receptor tyrosine phosphatase PTPRC (CD45) in T-cell acute lymphoblastic leukemia. *Blood*. May 10 2012;119(19):4476-9. doi:10.1182/blood-2011-09-379958

16. Hoshino A, Okada S, Yoshida K, et al. Abnormal hematopoiesis and autoimmunity in human subjects with germline IKZF1 mutations. *J Allergy Clin Immunol*. Jul 2017;140(1):223-231. doi:10.1016/j.jaci.2016.09.029

17. Kirstetter P, Thomas M. Ikaros is critical for B cell differentiation and function. *Eur J Immunol*. Mar 2002;32(3):720-30. doi:10.1002/1521-4141(200203)32:3<720::AID-IMMU720>3.0.CO;2-P

18. O'Brien S, Thomas RM, Wertheim GB, Zhang F, Shen H, Wells AD. Ikaros imposes a barrier to CD8+ T cell differentiation by restricting autocrine IL-2 production. *J Immunol*. Jun 1 2014;192(11):5118-29. doi:10.4049/jimmunol.1301992

19. Lutz-Nicoladoni C, Wolf D, Sopper S. Modulation of Immune Cell Functions by the E3 Ligase Cbl-b. *Front Oncol*. 2015;5:58. doi:10.3389/fonc.2015.00058

20. Zou Q, Jin J, Xiao Y, et al. T cell development involves TRAF3IP3-mediated ERK signaling in the Golgi. *J Exp Med*. Jul 27 2015;212(8):1323-36. doi:10.1084/jem.20150110

21. Dong S, Corre B, Nika K, Pellegrini S, Michel F. T cell receptor signal initiation induced by low-grade stimulation requires the cooperation of LAT in human T cells. *PLoS One*. Nov 30 2010;5(11):e15114. doi:10.1371/journal.pone.0015114

22. Sowd GA, Serrao E, Wang H, et al. A critical role for alternative polyadenylation factor CPSF6 in targeting HIV-1 integration to transcriptionally active chromatin. *Proc Natl Acad Sci U S A*. Feb 23 2016;113(8):E1054-63. doi:10.1073/pnas.1524213113

23. Wang Q, Wang C, Li N, et al. Condensin Smc4 promotes inflammatory innate immune response by epigenetically enhancing NEMO transcription. *J Autoimmun*. Aug 2018;92:67-76. doi:10.1016/j.jaut.2018.05.004

24. Bleilevens C, Soppert J, Hoffmann A, et al. Macrophage Migration Inhibitory Factor (MIF) Plasma Concentration in Critically Ill COVID-19 Patients: A Prospective Observational Study. *Diagnostics (Basel)*. Feb 17 2021;11(2)doi:10.3390/diagnostics11020332

25. Ruetsch C, Brglez V, Cremoni M, et al. Functional Exhaustion of Type I and II Interferons Production in Severe COVID-19 Patients. *Front Med (Lausanne)*. 2020;7:603961. doi:10.3389/fmed.2020.603961

26. Moore JB, June CH. Cytokine release syndrome in severe COVID-19. *Science*. May 1 2020;368(6490):473-474. doi:10.1126/science.abb8925

27. Solaro RJ, Warren CM, Scruggs SB. Why is it important to analyze the cardiac sarcomere subproteome? *Expert Rev Proteomics*. Jun 2010;7(3):311-4. doi:10.1586/epr.10.15

28. Poleshko A, Shah PP, Gupta M, et al. Genome-Nuclear Lamina Interactions Regulate Cardiac Stem Cell Lineage Restriction. *Cell*. Oct 19 2017;171(3):573-587 e14. doi:10.1016/j.cell.2017.09.018

29. McNally EM, Mestroni L. Dilated Cardiomyopathy: Genetic Determinants and Mechanisms. *Circ Res*. Sep 15 2017;121(7):731-748. doi:10.1161/CIRCRESAHA.116.309396

30. Razmara E, Garshasbi M. Whole-exome sequencing identifies R1279X of MYH6 gene to be associated with congenital heart disease. *BMC Cardiovasc Disord*. Jul 3 2018;18(1):137. doi:10.1186/s12872-018-0867-4

31. Jones PP, Guo W, Chen SRW. Control of cardiac ryanodine receptor by sarcoplasmic reticulum luminal Ca(2). *J Gen Physiol*. Sep 4 2017;149(9):867-875. doi:10.1085/jgp.201711805

32. Jiao Q, Bai Y, Akaike T, Takeshima H, Ishikawa Y, Minamisawa S. Sarcalumenin is essential for maintaining cardiac function during endurance exercise training. *Am J Physiol Heart Circ Physiol*. Aug 2009;297(2):H576-82. doi:10.1152/ajpheart.00946.2008

33. Napolitano C, Bloise R, Memmi M, Priori SG. Clinical utility gene card for: Catecholaminergic polymorphic ventricular tachycardia (CPVT). *Eur J Hum Genet*. Jan 2014;22(1)doi:10.1038/ejhg.2013.55

34. Haghighi K, Bidwell P, Kranias EG. Phospholamban interactome in cardiac contractility and survival: A new vision of an old friend. *J Mol Cell Cardiol*. Dec 2014;77:160-7. doi:10.1016/j.yjmcc.2014.10.005

35. Wu CI, Postema PG, Arbelo E, et al. SARS-CoV-2, COVID-19, and inherited arrhythmia syndromes. *Heart Rhythm*. Sep 2020;17(9):1456-1462. doi:10.1016/j.hrthm.2020.03.024

36. Cerrone M, Montnach J, Lin X, et al. Plakophilin-2 is required for transcription of genes that control calcium cycling and cardiac rhythm. *Nat Commun*. Jul 24 2017;8(1):106. doi:10.1038/s41467-017-00127-0

37. Benson MA, Tinsley CL, Waite AJ, et al. Ryanodine receptors are part of the myospryn complex in cardiac muscle. *Sci Rep*. Jul 24 2017;7(1):6312. doi:10.1038/s41598-017-06395-6

38. Bramante CT, Ingraham NE, Murray TA, et al. Metformin and risk of mortality in patients hospitalised with COVID-19: a retrospective cohort analysis. *Lancet Healthy Longev*. Jan 2021;2(1):e34-e41. doi:10.1016/S2666-7568(20)30033-7

39. Lou JS, Yan L, Bi CW, et al. Yu Ping Feng San reverses cisplatin-induced multi-drug resistance in lung cancer cells via regulating drug transporters and p62/TRAF6 signalling. *Sci Rep*. Aug 25 2016;6:31926. doi:10.1038/srep31926

40. Shen JJ, Lin CJ, Huang JL, Hsieh KH, Kuo ML. The effect of liu-wei-di-huang wan on cytokine gene expression from human peripheral blood lymphocytes. *Am J Chin Med*. 2003;31(2):247-57. doi:10.1142/S0192415X03000886

41. Shih YS, Tsai CH, Li TC, et al. The effect of Xiang-Sha-Liu-Jun-Zi tang (XSLJZT) on irritable bowel syndrome: A randomized, double-blind, placebo-controlled trial. *J Ethnopharmacol*. Jun 28 2019;238:111889. doi:10.1016/j.jep.2019.111889
